# A Nonsense Variant in *CCDC65* Gene Causes Respiratory Failure Associated with Increased Lamb Mortality in French Lacaune Dairy Sheep

**DOI:** 10.3390/genes13010045

**Published:** 2021-12-24

**Authors:** Maxime Ben Braiek, Carole Moreno-Romieux, Charlotte Allain, Philippe Bardou, Arnaud Bordes, Frédéric Debat, Cord Drögemüller, Florence Plisson-Petit, David Portes, Julien Sarry, Némuel Tadi, Florent Woloszyn, Stéphane Fabre

**Affiliations:** 1 GenPhySE, Université de Toulouse, Institut National de Recherche Pour L’agriculture, L’alimentation et l’environnement (INRAE), Ecole Nationale Vétérinaire de Toulouse (ENVT), 31326 Castanet-Tolosan, France; maxime.ben-braiek@inrae.fr (M.B.B.); carole.moreno-romieux@inrae.fr (C.M.-R.); arnaud.bordes@inrae.fr (A.B.); frederic.debat@inrae.fr (F.D.); florence.plisson-petit@inrae.fr (F.P.-P.); julien.sarry@inrae.fr (J.S.); nemuel.tadi@inrae.fr (N.T.); florent.woloszyn@inrae.fr (F.W.); 2 UE Domaine de La Fage, Institut National de Recherche Pour L’agriculture, L’alimentation et L’environnement (INRAE), 12250 Saint-Jean et Saint-Paul, France; charlotte.allain@inrae.fr (C.A.); david.portes@inrae.fr (D.P.); 3 Sigenae, Institut National de Recherche Pour L’agriculture, L’alimentation et L’environnement (INRAE), 31326 Castanet-Tolosan, France; philippe.bardou@inrae.fr; 4 Institute of Genetics, Vetsuisse Faculty, University of Bern, 3012 Bern, Switzerland; cord.droegemueller@vetsuisse.unibe.ch

**Keywords:** homozygous haplotype deficiency, LDHH6, CCDC65, juvenile lethal mutation, genetic disorder, primary cilia dyskinesia (PCD), ciliopathy, development, sheep

## Abstract

We recently demonstrated that the Lacaune deficient homozygous haplotype 6 (LDHH6) potentially hosts a recessive perinatal lethal mutation in Lacaune dairy sheep mapped on OAR3. In the present study, we have analyzed the whole-genome sequences of two Lacaune ram heterozygous carriers of LDHH6. After variant calling and filtering against the variants of 86 non-carrier rams, we have identified a single nucleotide variant (SNV) in the two LDHH6 carriers whose variant allele induced a premature stop codon (p.Glu111*) in the *Coiled-Coil Domain Containing 65* (*CCDC65*) gene. CCDC65 is involved in the assembly of the nexin-dynein regulatory complex for the formation of microtubules in ciliated cells. In order to identify the phenotype in homozygous sheep, we generated at-risk matings (*n* = 17) between rams and ewes heterozygous for the candidate variant in *CCDC65*. A total of 16 lambs were born alive with five genotyped as homozygous carriers. The homozygous lambs suffered from respiratory problems, and four of them died within the first month of life. At necropsy, we observed a broad hepatization of lung lobes possibly induced by infectious pneumonia. The management of this lethal recessive allele (frequency of 0.06) through reasoned mating in the Lacaune sheep selection schemes could reduce lamb mortality by 2%.

## 1. Introduction

Health and animal welfare are major concerns in livestock populations with important economic repercussions. During the last few decades, mortality in the first month of life remains high around 15–20% in sheep and most of this mortality occurs within the first three days of postnatal life [1,2]. The risk factors for lamb mortality are mainly due to parameters depending on the mother (nutrition, mothering ability, health status, parturition conditions), the lamb itself (low birth weight, vigor, colostrum intake, congenital malformations) and/or their environment (extreme weather, predation, infectious diseases) [1,3].

Although adequate farm environment and management practices could favor lamb viability (reviewed in Dwyer et al. [1]), it would be possible to act on the genetic aspect of lamb survival. However, the QTL approach for mapping genetic variants/loci affecting this trait is difficult due to low heritability (h^2^ < 0.1) and multifactorial nature of lamb survival or lamb birthweight (i.e., best proxy for lamb survival) [1,4,5,6]. Nevertheless, thanks to the use of high throughput genomic tools (single nucleotide polymorphism arrays, whole-genome sequences), the study of genetic disorders and analysis of associated data have successfully led the identification of many causal variants affecting the viability of young animals [7].

Nowadays, two main approaches are used to identify and characterize causal variants associated with genetic disorders. The first one is the homozygosity-mapping based on a case-control approach using only few biological samples from affected and non-affected animals [8,9,10]. In this pioneering use of homozygosity-mapping using large number of SNP array in cattle, Charlier et al. have identified three causal variants responsible for *congenital muscular dystony* types 1 [OMIA 001450-9913] and 2 [OMIA 001451-9913] in Belgian Blue cattle and *ichthyosis fetalis* [OMIA 002238-9913] in Italian Chianina cattle [8]. In sheep, this approach was used successfully for the first time in the *microphthalmia* [OMIA 000649-9940] in Texel [11]. Since the last few decades, this method is efficient to map genetic defects and their associated variants as shown by the growing number of discovered causal variants referenced in the OMIA: Online Mendelian Inheritance in Animal database (https://www.omia.org/; accessed 2 September 2021). As example, with this approach, several pathogenic variants were identified causing *junctional epidermolysis bullosa* in cattle [12,13] and sheep [14,15,16] and the affected animals died shortly after birth due to skin disorders (injuries on limb extremities, mucous membranes). However, when mutations affect viability of developing embryos during gestation, phenotype or biological samples are not available. Thus, these mutations are more efficiently detectable by a second approach using reverse genetic screens. This approach is based on the availability of large sets of SNP array genotyped animals provided by genomic selection for example [17,18]. Reverse screens identified haplotypes for which homozygous carrier animals are absent or significantly less observed when compared to the expected number based on haplotype frequency. In cattle, the original works of VanRaden et al. [17] and Fritz et al. [18] using reverse genetic screen followed by whole-genome sequence analyses have successfully identified candidate causal variants in linkage disequilibrium with haplotypes with significant partial or total deficit in homozygous animals. Going on with this approach, numerous embryonic lethal mutations were identified in Holstein and Jersey breeds altering *APAF1* [19], *SMC2* [20,21], *GART* [18], *TFB1M* [22], *SDE2* [23], *CENPU* [24] and *CWC15* [25] genes. Additionally, neonatal/juvenile lethal mutations associated with homozygous haplotype deficiency were also identified in Ayshire and Braunvieh breeds affecting *UBE3B* [20] and *TUBD1* [26] genes.

Recently, with the implementation of genomic selection in small ruminants [27] and the availability of large genotyping datasets, we have identified homozygous deficient haplotypes segregating in the Lacaune dairy sheep population by a reverse genetic screen method [28]. We have identified 11 haplotypes with significant deficit in homozygous animals, ranging from 79 to 100%. Some of them were associated with fertility loss at artificial insemination, and/or with increased stillbirth rate. We therefore assumed that these haplotypes are likely to host recessive mutations causing embryonic/fetal or perinatal lethality, respectively. Among these haplotypes, LDHH6 (Lacaune deficient homozygous haplotype 6) [OMIA 002342-9940] was the most frequent (12.1% of heterozygous carriers, located on OAR3:146.2–147.9 Mb on Oar_rambouillet_v1.0) and presented a homozygous deficit of 96% (three homozygous animals were observed, whereas 72 animals were expected; *p* = 3.5 × 10^−27^). LDHH6 showed an increased stillbirth rate in at-risk matings between carrier rams and daughters of carrier rams when compared to safe matings. We have previously reported several candidate genes in the LDHH6 region (*WNT1*, *CCDC65* and *PFKM* genes), but the underlying causative variant is still unknown [28].

In the present study, we have taken advantage of whole-genome sequence data to identify the causal variant associated with the LDHH6 haplotype, studied the segregation of the variant in several sheep breeds and validated the lethal effect of the proposed causal variant by generating at-risk matings.

## 2. Materials and Methods

### 2.1. Sequencing Data

For variant calling, publicly available data of 88 ovine short read Illumina HiSeq whole genome sequences (WGS) from 14 breeds generated in various INRAE and Teagasc research projects were used. Among them, 24 WGS were obtained from dairy Lacaune sheep, and also genotyped with the OvineSNP50 Beadchip from Illumina in the framework of Lacaune dairy sheep genomic selection program [27]. A description of the different breeds and all the accession numbers of sequencing raw data are available in Supplementary Table S1. 

### 2.2. WGS Variant Calling and Annotation

Reads mapping and variants calling were performed using Nextflow v20.10.0 and Sarek pipeline v2.6.1 [29]. Genome Analysis Toolkit (GATK) best practices were followed as implemented in the Sarek pipeline. In this project, the following steps were performed: aligning the reads with BWA v0.7.17-r1188 [30] against the ovine genome assembly Oar_rambouillet_v1.0 (GCF_002742125.1), marking duplicate reads (MarkDuplicates), base quality recalibration (BQSR) and calling germline small variants (HaplotypeCaller in GVCF mode) with GATK v4.1.7.0 [31], annotation of small variants with SnpEff v4.3t [32] and quality control with MultiQC v1.8 [33].

### 2.3. Identification of Candidate Causal Variants

Based on OvineSNP50 Beadchip genotyping, two Lacaune animals were detected as LDHH6 heterozygous carriers (others as non-carriers) among the 24 sequenced Lacaune genomes [28]. All SNPs, small insertion and deletion variants located within the LDHH6 region extended by 1 Mb from each side were extracted from OAR3 (Oar_rambouillet_v1.0; NC_040254.1:145,243,481-148,946,399pb) using SnpSift Filter [32]. The selection filter of candidate polymorphisms was as follows: (i) variant allele compared to the Rambouillet reference genome; (ii) in the heterozygote state in the 2 heterozygous LDHH6-carriers Lacaune sheep; and (iii) in the reference homozygous state in all LDHH6-non carriers Lacaune sheep and in other non-related breeds. These variants were checked manually using the Integrative Genomics Viewer (IGV) [34] to confirm the bioinformatics variant calling prior to further investigation.

### 2.4. Biological Samples and CCDC65-Specific Genotyping Assay

Blood samples (3 ml) were collected by jugular vein puncture with the Venoject system containing EDTA (Terumo, Tokyo, Japan) and directly stored at 4 °C or −20 °C depending on further use. Ear samples were obtained with a tissue sampling unit (TSU, Allflex Europe, Vitré, France) taking an ear punch (1 mm^3^) directly placed in the TSU storage buffer at 4 °C. Ear biopsies were placed twice consecutively in 180 μL of 50 mM NaOH, heated 10 min at 95 °C, neutralized with 20 μL of 1 M HCl, and then vortexed during 10 s. Part of the blood samples was used for extraction of genomic DNA as described in Bodin et al. [35]. All other samples were used for direct genotyping without DNA purification on whole blood or on neutralized NaOH treatment solution of ear biopsies [36].

Among the candidate polymorphisms within the LDHH6 region, the potential *CCDC65* causal variant (Oar_rambouillet_v1.0, NC_040254.1:g.147,207,999C>A; XM_004006389.4:c.521G>T) was genotyped either by RFLP or PACE (PCR allele competitive extension) analysis. RFLP was resolved on 2% agarose gel using BsaJI restriction enzyme (News England Biolabs, Ipswich, MA, USA) after either a first step of Terra PCR Direct Polymerase Mix amplification (Takara Bio, Kusatsu, Japan) using 1 µL of total blood (or 5 µL ear biopsy solution), or a PCR using GoTaq Flexi DNA Polymerase (Promega, Madison, WI, USA) with 50 ng of purified DNA. The following amplification primers (forward 5′-GAGCTGCGTGTGTAAGATGA-3′ and reverse 5′-CCTCCAGCTCCATGTTGTAA-3′) were designed using Primer3Plus software [37]. PCR for RFLP was performed on an ABI2720 thermocycler (Applied Biosystems, Waltham, MA, USA) with the following conditions: 5 min at 94 °C, 35 cycles of 30 s at 94 °C, 30 s at 58 °C and 30 s at 72 °C, followed by 5 min final extension at 72 °C. Fluorescent PACE analysis was done with 15 ng of purified DNA using the PACE-IR 2x Genotyping Master mix (3CR Bioscience) in the presence of 12 µM of a mix of extended allele specific forward primers (5′-GAAGGTGACCAAGTTCATGCTGGACCTGTCAGAAGCCGAGG-3′ and 5′-GAAGGTGACCAAGTTCATGCTGGACCTGTCAGAAGCCGAGT-3′) and 30 µM of common reverse primer (5′-AGGGCGTGGGCGTGCTGCT-3′) in a final volume of 10 μL. The touch-down PCR amplification condition was 15 min at 94 °C for the hot-start activation, 10 cycles of 20 s at 94 °C, 54–62 °C for 60 s (dropping 0.8 °C per cycle), then 36 cycles of 20 s at 94 °C and 60 s at 54 °C performed on an ABI9700 thermocycler followed by a final point read of the fluorescence on an ABI QuantStudio 6 real-time PCR system and using the QuantStudio software 1.3 (Applied Biosystems). The accuracy of the genotyping was validated by Sanger sequencing on few samples.

The presence of the *CCDC65* variant was checked in a DNA set of the 2021 cohort of 2952 Lacaune male lamb candidates for genomic selection. DNA was extracted by Labogena (http://www.labogena.fr, accessed on 2 September 2021) for low-density SNP chip genotyping (SheepLD v.3, on behalf the dairy Lacaune breed industry) and LDHH6 status was established as previously described [28]. A DNA diversity panel of 872 animals from 25 French sheep breeds [38] and 8 Swiss sheep breeds [39] was also specifically genotyped for the *CCDC65* variant.

### 2.5. Generation of Homozygous Lambs

Dairy Lacaune ewes (*n* = 245) from two INRAE experimental flocks (Langlade and La Fage, agreement numbers: D3142901 and A312031, respectively) were genotyped for the *CCDC65* variant. Heterozygous ewes (*n* = 17) were artificially inseminated (AI) with fresh semen from heterozygous carrier rams (*n* = 3) selected among the genotyped animals for genomic selection with a known status at the LDHH6 haplotype [28] and further genotyped as heterozygous at the *CCDC65* locus. An ultrasound diagnosis of gestation was realized between 45 and 60 days after AI. Lambs were weighted at birth and 15 days after birth. Ear biopsies from newborn lambs (TSU Allflex) were collected for *CCDC65* genotyping at the same time of the mandatory electronic ear tag identification.

### 2.6. Ethics Statement

All experimental procedures were approved (approval numbers 01171.02 and 752056.00) by the French Ministry of Teaching and Scientific Research and local ethical committee C2EA-115 (Science and Animal Health) in accordance with the European Union Directive 2010/63/EU on the protection of animals used for scientific purposes.

## 3. Results

### 3.1. Screening of WGS Data Identifies a Nonsense Variant in CCDC65 Gene Associated with LDHH6

To identify the putative causal variant hosted by the LDHH6 haplotype, we considered biallelic polymorphisms (SNPs and InDels) proven from 88 ovine WGS containing 24 Lacaune sequences and, among them, two LDHH6 heterozygous carriers. Variant search analysis and annotations were deliberately limited to the LDHH6 region (OAR3: 146,243,481–147,946,399 pb) extended by 1 Mb from each side. In this 3.7 Mb region, we detected 53,632 polymorphisms with a quality score > 30. After filtering, we identified 11 SNPs only present in a heterozygous state in the genome of the two LDHH6 heterozygous carriers (Table 1).

**Figure 1:**
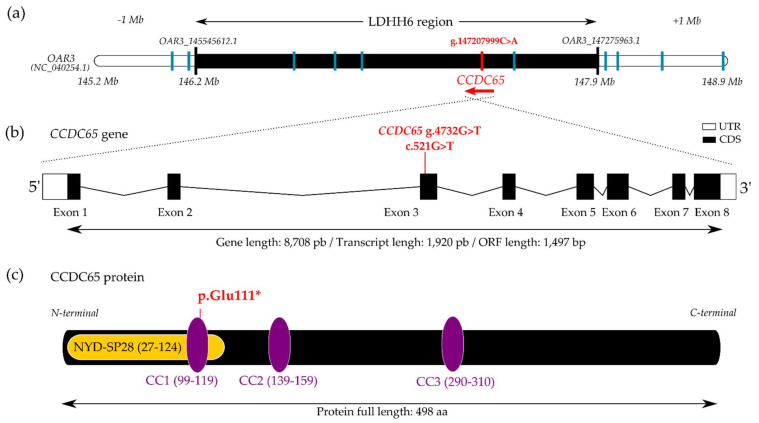
Nonsense variant in *CCDC65* gene associated with the LDHH6 lethal haplotype. (**a**) LDHH6 haplotype (NC_040254.1:OAR3:146,243,481pb-147,946,399pb) extended by 1 Mb from each side. The black bar indicates the limits of LDHH6 haplotype with the first and last markers (Illumina OvineSNP50 OAR3_145545612.1 and OAR3_147275963.1). Vertical lines indicate the positions of the candidate causal SNV in *CCDC65* (red) and 10 other SNVs fully associated with LDHH6 (blue); (**b**) *CCDC65* gene structure and localization of the g.4732G > T (GeneID:101104220)/c.521G > T (XM_004006389.4) variant in the third exon (UTR: untranslated region, CDS: coding sequence); (**c**) schematic representation of CCDC65 protein (XP_004006438.1) with one N-terminal ‘NYD-SP28’ (Pfam PF14772) and three coiled-coil (CC) domains (UniProtKB-W5QCQ9), and position of the premature C-terminal residue, pGlu111.

Among those SNPs, only one was predicted to highly alter gene function. This SNV (NC_040254.1:g.147,207,999C > A; XM_004006389.4:c.521G > T) leads to a nonsense variant located in exon 3 of the *Coiled-Coil Domain Containing 65* (*CCDC65*) gene (Figure 1a,b). The variant should result in a premature stop codon and a truncated protein in position 111 (XP_004006438.1:p.Glu111*) while the entire translated CCDC65 protein is composed of 498 amino acids (Figure 1c). The variant localizes in the first Coiled-Coil (CC) domain in a highly conserved region of the protein between mammal species and even in algae (*Chlamydomonas reinhardtii*) where the role of CCDC65 was originally studied [40,41,42,43] (Supplementary Figure S1). The resulting truncated protein has kept the major part of the NYD-SP28 domain shared with CCDC164 also part of the N-DRC [44] but has lost two CC domains known to be implicated in protein–protein interaction [42,43].

### 3.2. Variant Association with LDHH6 and Population Estimation of Allele Frequency

In order to provide additional evidence in favor of the *CCDC65* polymorphism as the causal variant, we have genotyped the c.521G > T SNP in a cohort of 2952 Lacaune lambs with known status at the LDHH6 locus (Supplementary Figure S2). The contingency table indicated a clear association between the LDHH6 status and the nonsense variant in *CCDC65* (Table 2, Fischer’s exact test *p* < 0.0001, without the homozygous carrier individual). Based on this genotyping, the c.521T variant allele frequency was calculated at 7%, and, as expected, the distribution of genotypes was not consistent with the Hardy–Weinberg equilibrium (Chi-square test, *p* < 0.001).

**Figure 2:**
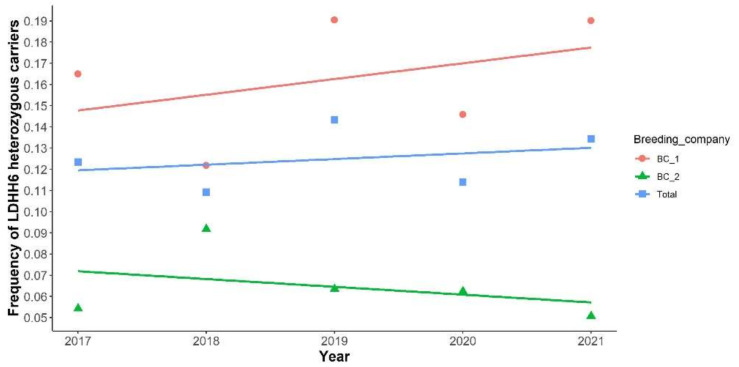
Evolution of LDHH6 heterozygous carrier frequency between 2017 and 2021 in dairy Lacaune male lambs. The frequency is indicated either for all candidates to genomic selection (Total) or depending on the breeding company (BC_1 or 2).

Since 2017, all Lacaune male lamb candidates for genomic selection were genotyped on low-density sheep (SheepLD chip) between one and five months of age, with a good representation of the genetic diversity in the selection schemes. In the Lacaune dairy breed, the population consists of two subpopulations with separate selection schemes conducted by two breeding companies. Overall, LDHH6 heterozygous carrier frequency observed is stable in the population between 2017 and 2021. Nevertheless, the frequency of carriers was two-fold higher for breeding company 1 compared to breeding company 2 (Figure 2).

### 3.3. Generation of At-Risk Matings to Obtain Homozygote Lambs

**Figure 3:**
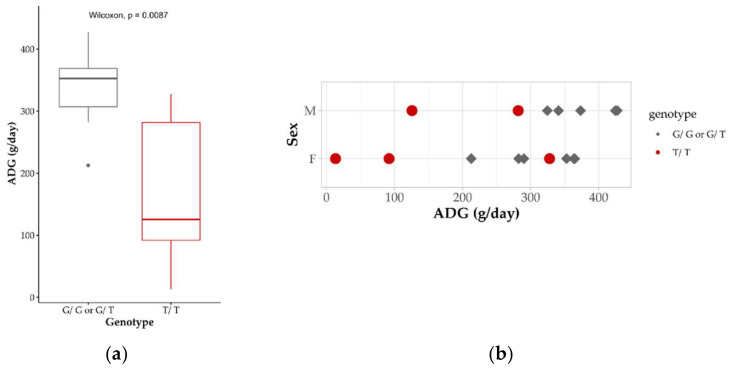
*CCDC65* genotype effect on average daily gain during the 0–15 day period. Mean ADG (g/day) according to *CCDC65* c.521G > T genotype (**a**) and ADG distribution by sex (M: male; F: female) and genotype (**b**).

The genotyping of the *CCDC65* variant in two experimental flocks of dairy Lacaune ewes (*n* = 245) enabled us to identify 17 heterozygous carriers. These ewes were inseminated with fresh semen from heterozygous carrier rams to generate at-risk matings. Forty-five days after AI, 11 ewes were diagnosed as pregnant by ultrasonography. This corresponded to an AI success of 65%, in line with the average AI success of 69% recorded in the whole dairy Lacaune population [28]. Gestation length was recorded as normal (146 ± 2 days), and no abortion or stillborn was observed. At the end of gestation, 16 lambs were born with 7 males and 9 females. For each lamb, an ear punch was collected for genotyping of the c.521G > T *CCDC65* variant. Five lambs were genotyped homozygous non-carriers, 6 were heterozygous and 5 were homozygous carriers, consistent with the Hardy–Weinberg equilibrium (Chi-square test, *p* = 0.317). All lambs were weighted at birth (males: 4.9 ± 1.0 kg, females: 3.9 ± 0.6 kg), and no significant difference of birthweight was observed between genotypes. A second weighing was carried out at day 15 and average daily gain (ADG) was calculated on the 0–15 day period. Based on Wilcoxon’s non-parametric test, the homozygous carrier (T/T) showed a significant lower ADG compared to the other lambs (G/G or G/T) (Figure 3).

Clinical examination revealed respiratory problems, such as tachypnea and a runny nose for 4 over 5 T/T lambs from the first days of life. Despite an appropriate veterinary treatment for suspected pulmonary infectious diseases (based on glucocorticoids and antibiotics administration), the recurrent respiratory distress resulted in the declining of general body condition (as attested by the lower ADG) leading to the natural death or euthanasia of the affected lambs between 15 and 25 days after birth. Only one T/T female lamb with the highest ADG (327.8 g/day during the 0–15 day period) has exceeded the weaning age with light respiratory syndrome and was sold to a sheep fattener.

Additionally, the previous analysis of the genomic selection cohort has allowed to identify a homozygous animal for the *CCDC65* variant (Table 2). This male lamb was present in a breeding center, and we obtained the information that he received an adapted veterinary treatment several times due to respiratory problems. The lamb finally died at the age of five and a half months. Overall, these observations have confirmed the hypothesis of a deleterious recessive *CCDC65* variant almost perfectly associated with LDHH6.

**Figure 4:**
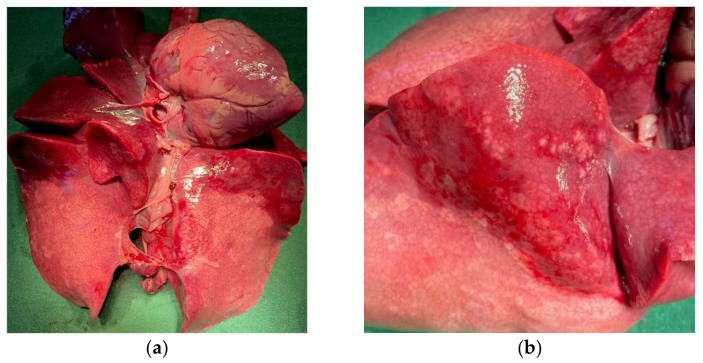
*CCDC65* homozygous affected lamb with lung lesions. (**a**) heart-pulmonary system of an affected homozygous lamb; (**b**) magnification of hepatized lobes.

An autopsy was performed on the four deceased T/T lambs. A broad hepatization of lungs probably linked to an infectious pneumonia was particularly observed (Figure 4).

### 3.4. Occurence of the CCDC65 Variant in Further Populations

An ovine DNA diversity panel of 25 French and eight Swiss sheep breeds representing 872 animals was checked for the segregation of the c.521G > T variant in *CCDC65* (Table 3). Initially shown in the French Lacaune dairy sheep, *CCDC65* genotyping has also revealed its occurrence in the French Lacaune meat strain and in a Lacaune dairy population reared in Switzerland. Moreover, two heterozygous animals were shown in the Blanche du Massif Central (BMC) breed. All the other tested French and Swiss breeds were non-carriers of the T allele.

## 4. Discussion

We have previously reported that LDHH6 [OMIA 002342-9940] was the most significant haplotype in deficit of homozygous animals in French Lacaune dairy sheep. Analyses based on at-risk matings between LDHH6 heterozygous carriers have shown an increase in stillbirth rate, suggesting that this haplotype harbored a harmful recessive mutation [28]. Using sheep whole-genome sequences, the present study identified a causal variant as the g.147,207,999C > A substitution on ovine chromosome 3 linked to the LDHH6 haplotype, and this SNV corresponds to the c.521G > T nonsense variation in the *CCDC65* gene introducing a premature stop codon (p.Glu111*).

In the LDHH6 region, we have previously highlighted three candidate genes, *WNT1*, *CCDC65*, and *PFKM*, whose knock-out models in mice fitted well with perinatal, neonatal or preweaning lethality [28]. Interestingly, variant analysis from the whole genome sequence of LDHH6 heterozygous carriers has revealed candidate variants in ovine *CCDC65*, but not in *WNT1* or *PFKM* or in close vicinity of these two genes. Moreover, among all candidate polymorphisms, the SNP detected in the exon 3 of *CCDC65* was the most obvious candidate since it was the only one predicted to strongly alter the protein function by creating a premature stop codon allowing to synthesize only the first N-terminal quarter of the protein.

In humans, pathogenic variants in *CCDC65* cause Primary Cilia Dyskinesia (PCD), a genetic disorder with autosomal recessive inheritance (CILD27, OMIM#615504). The protein is involved in the assembly of the axonemal nexin-dynein regulatory complex [45]. Axoneme is the axial motor part of cilias composed of nine doublets of microtubules located in the periphery and one central pair complex [40]. Each outer doublet is composed of two tubules (A and B) and is associated with several complexes: outer and inner dynein arms (ODAs and IDs), Nexin-Dynein Regulatory Complex (N-DRC) and Radial Spoke (RS) [40,41,42,43,46]. In particular, the N-DRC is important for the sliding between adjacent outer doublet microtubules to allow cilia motility. The complex is composed of at least 11 subunits shown in *C. reinhardtii* and conserved among mammals [40,41,42,43]. Among them, variants in one of the three genes: *CCDC164* (OMIM #615294 [47]), *CCDC65* (OMIM #615504 [44,48]) or *GAS8* (OMIM #616726 [49]) have been reported to cause PCD. Bower et al. have demonstrated that the absence of CCDC65 subunit destabilized the association with CCDC164 and GAS8 [45]. Indeed, CCDC65 forms a base plate for N-DRC. Alteration in N-DRC have big consequences for cilia beating leading to the respiratory problems. These three subunits are important for cilia and flagella motility and mucus clearance to eject pathogenic organisms from respiratory tracts. In human, loss-of-function mutations in *CCDC65* genes in PCD patients have been identified and are associated with recurrent infectious diseases of the ENT (ear nose throat) sphere as bronchitis, sinusitis, and/or otitis [44,48]. The above clinical profiles are well in line with our observations made in lambs homozygous for the c.521G > T variant in *CCDC65*. Homozygous lambs have recurrent neonatal respiratory problems. At the autopsy, we particularly observed a broad hepatization of lung lobes resembling infectious pneumonia also associated with growth delay [50]. We hypothesize that, due to the loss of Coiled-Coil domains, the truncated CCDC65 leads to the destabilization of the N-DRC, abnormal cilia beating and impairs the mucociliary clearance resulting in airways obstruction by mucus loaded with pathogens (bacteria, mycoplasma, virus). Pathogens load could depend on the breeding sanitary conditions, individual immunity and veterinary treatment applied, which can explain that the lethality can occur between perinatal and juvenile stages. This is to be connected to the previous observation of a partial homozygous deficit for LDHH6 at the age of genotyping (72 expected and 3 observed) [28]. Indeed, Lacaune candidate rams for genomic selection are genotyped on average at 3 months of age using LD SNP chip indicating that some LDHH6/*CCDC65* homozygous lambs have exceeded this age as observed for a female born from our at-risk mating, and a male in a breeding center exceding 5 months of age.

Interestingly, a respiratory syndrome is also reported in cattle for the homozygous deficient Braunvieh haplotype 2 (BH2) [OMIA 001939-9913] associated with perinatal and juvenile mortality due to the missense variant (p.His210Arg) in *TUBD1* (*tubulin delta 1*) that also disorganizes the microtubules in airway cilia [26]. Additionally, a stop-gain variant in *CCDC39* (p.Arg96*) [OMIA 001540-9615], also essential for the assembly of inner dynein arms [51,52], was found in Bobtail dogs associated with PCD suffering from chronic airway diseases [51].

Based on the sheep gene atlas (http://biogps.org/sheepatlas/; accessed 2 September 2021), CCDC65 is highly expressed in tissues and organs hosting ciliated or flagellated cells as lungs, fallopian tube and testis [53]. The high expression in testis raises the question on the impact of the *CCDC65* variant on sperm motility, knowing that the first characterization of the protein was made in human sperm tail [54] and several mutations causing PCD lead to sperm defects [55]. The three homozygous male lambs we had access to died before puberty, preventing us from getting the semen. We then evaluated the impact of the mutation at the heterozygous state based on semen quality records from artificial insemination center. Semen quality is controlled for each ejaculate according to volume, concentration and sperm motility [56]. However, no significant effect of the mutation was demonstrated for the parameters recorded (Supplementary Table S2).

By genotyping the complete 2021 cohort of candidate lambs for genomic selection, we have validated an almost perfect association between LDHH6 status and alleles at the *CCDC65* variant (Table 2). Only 12 among 2952 animals do not match with the expectations (described in Supplementary Figure S3). Indeed, nine LDHH6 non-carriers were heterozygous carriers for the variant allele. Specific focus on the haplotypes of these animals have revealed shorter recombinant versions of the LDHH6 haplotypes (between 4 and 26 markers out of 27 markers composing the LDHH6 haplotype). We also identify three LDHH6 heterozygous carriers which do not carry the *CCDC65* variant. This discrepancy could be attributed to errors from SNP array genotyping, phasing and/or imputation.

Diversity analysis revealed the segregation of the *CCDC65* c.521G > T variant not only in the Lacaune dairy population but also in the Lacaune meat and Blanche du Massif Central (BMC) populations. Additionally, we found the *CCDC65* variant in the Ensembl variant database (rs1085624756) compiling the whole genome sequences from the International Sheep Genome Consortium (448 animals from 58 breeds all over the world). The rs1085624756 variant is only present in a Lacaune dairy animal (LRLACU000000000084). The Lacaune sheep population has a complex history with the creation of two lines, one for meat and one for dairy purposes, with four independent selection schemes depending on two breeding companies. Intriguingly, the LDHH6 carrier frequency is 2.5 fold higher in one breeding company compared to the other one (0.16 vs. 0.06). In livestock, deleterious alleles could be associated with better performances when heterozygous to explain their maintaining in the populations [7,57,58]. However, no heterozygous advantage has been identified on milk selected traits in the Lacaune population for LDHH6 heterozygous carriers [28], and the two breeding companies have similar selection objectives on milk traits. Thus, the difference observed in the *CCDC65* allele frequency between the two breeding companies could be explained by genetic drift and the use of very influential carriers. The segregation of the *CCDC65* variant in both dairy and meat strains of Lacaune sheep indicates that the mutation event predates the creation of the two lineages. Moreover, the variant is also segregating in the BMC breed. Population structure analyses revealed that Lacaune and BMC, both originating from the Massif Central in France, shared the same origin within the Southern European sheep populations that may explain the segregation of identical alleles in both populations [38]. Interestingly, these two breeds also share another important variant in the *BMP15* gene controling prolificacy [36]. The presence of the variant in Swiss Lacaune could be explained by imports of French Lacaune reproducers. However, with the number of carriers detected in our analysis (10 over 37), it is important to check the mutation frequency in the overall Swiss Lacaune population because the risk of generating homozygous lambs is quite high.

## 5. Conclusions

This is the first report of the identification of a recessive lethal variant in sheep through a reverse genetic screen method using hightroughput genotyping completed by whole genome sequencing of few informative animals. The present study identifies a causal recessive nonsense variant in *CCDC65* gene linked to the homozygous deficient haplotype named LDHH6 and associated with rearing loss in Lacaune dairy sheep. The loss-of-function mutation in *CCDC65* is likely to cause a *primary cilia dyskinesia* syndrome similar to *CCDC65*-related ciliopathies in humans. Homozygous affected lambs suffer from respiratory defects predisposing infectious pneumonia with a fatal ending at a young age. Based on the LDHH6/*CCDC65* allele frequency of around 0.06 and a mortality lamb rate at 15% (recorded on 804,577 matings, described in [28]), we estimate that 1/50 (2%) of dead lambs is homozygous for the *CCDC65* variant. Consequently, management of this causal variant in the Lacaune sheep selection schemes through reasoned mating of carrier rams and ewes is important to improve health, welfare and overall lamb viability.

## Figures and Tables

**Table 1:** Candidate SNPs, locations and functional annotations.

Position	Ref/Alt	Quality Score	Location Annotation	Functional Consequence ^a^
145,928,967	C/G	347.3	Intergenic, downstream of *FAM186A*	Modifier
146,173,708	G/A	396.0	Intergenic, upstream of *ASIC1*	Modifier
146,566,556	C/T	133.1	Intronic, *FAM186B*	Modifier
146,718,479	G/A	272.7	Intronic, *SPATS2*	Modifier
146,809,812	T/C	604.4	Intronic, *DNAJC22*	Modifier
147,207,999	C/A	506.7	Exonic, *CCDC65* (c.521G > T)	High, stop-gain (p.Glu111*)
147,345,297	G/A	192.3	Intergenic, upstream of *TEX49*	Modifier
148,189,184	A/G	596.7	Intergenic	Modifier
148,212,194	A/C	181.7	Intergenic	Modifier
148,417,713	A/G	545.8	Intergenic, downstream of *ZNF641*	Modifier
148,904,267	G/A	439.1	Intronic, *HDAC7*	Modifier

^a^ variant annotation and effect predicted by SnpEff [32].

**Table 2:** Contingency table between LDHH6 status and genotype at c.521G > T in *CCDC65*.

Genotype	+/+	LDHH6/+	LDHH6/LDHH6	Total
G/G	2540	3	0	2543
G/T	9	399	0	408
T/T	0	0	1	1
Total	2549	402	1	2952

+/+: non-carriers; LDHH6/+: heterozygous carriers and LDHH6/LDHH6: homozygous carriers.

**Table 3:** *CCDC65* c.521G > T genotype distribution from a DNA diversity panel of French (FR) and Swiss (CH) ovine breeds.

Breed	Total	Genotype		Breed	Total	Genotype	
		G/G	G/T			G/G	G/T
Berrichon du Cher (FR)	30	30		Mouton Vendéen (FR)	30	30	
Blanche du Massif Central (FR)	31	29	2	Noir du Velay (FR)	28	28	
Causse du Lot (FR)	32	32		Préalpes du sud (FR)	27	27	
Charmoise (FR)	31	31		Rava (FR)	29	29	
Charollais (FR)	30	30		Romane (FR)	30	30	
Corse (FR)	30	30		Romanov (FR)	26	26	
East Friesian (CH)	18	18		Rouge de l’Ouest (FR)	30	30	
Engadine Red (CH)	3	3		Roussin (FR)	30	30	
Ile de France (FR)	28	28		Saaser Mutte (CH)	10	10	
Lacaune (meat) (FR)	45	43	2	Suffolk (FR)	29	29	
Lacaune (milk) (FR)	40	37	3	Swiss Mirror (CH)	11	11	
Lacaune (milk) (CH)	37	27	10	Swiss White Alpine (CH)	14	14	
Limousine (FR)	30	30		Tarasconnaise (FR)	33	33	
Manech tête rousse (FR)	29	29		Texel (FR)	27	27	
Martinik (FR)	23	23		Valais Blacknose (CH)	14	14	
Merinos d’Arles (FR)	27	27		Valais Red (CH)	13	13	
Mourerous (FR)	27	27					
				Total	872	855	17

## Data Availability

The WGS data used in this study are publicly available, accession numbers are described in Table S1.

## Supplementary Materials

The following are available online at https://www.mdpi.com/article/10.3390/genes13010045/s1, Table S1: Accession numbers of the 88 whole-genome sequences used in the analysis, Table S2. Semen parameters of dairy Lacaune rams from artificial insemination center, Figure S1. CCDC65 protein multiple sequence alignment, Figure S2. Representative genotyping results for c.521G > T variant in *CCDC65,* Figure S3. Haplotypes of the LDHH6 region for 12 animals showing mismatch with the *CCDC65* SNV.
