# Supplementary material for: A Nonsense Variant in CCDC65 Gene Causes Respiratory Failure Associated with Increased Lamb Mortality in French Lacaune Dairy Sheep
Source: Genes (Basel). 2021 Dec 24;13(1):45. doi: 10.3390/genes13010045 (PMC8774411; doi:10.3390/genes13010045)
Supplement: Supplementary file 1 [file genes-13-00045-s001.zip › genes-1490931-supplementary.pdf]

Supplementary Materials

Table S1. Accession numbers of the 88 whole-genome sequences used in the analysis

Breed	Number of animals	ENA run accession	EBI project accession	Associated references
Dairy Lacaune	24	ERR3276357; ERR3276358; ERR3276359; ERR3276360; ERR3276361; ERR3276362; ERR3276363; ERR3276364; ERR3276365; ERR3276368; ERR3276369; ERR3276370; ERR3276371; ERR3276372; **ERR3276373***; ERR3276374; ERR3276375; **ERR3276376***; ERR3276377; ERR3276378; ERR3276379<br>ERR968423; ERR968424; ERR968425	PRJEB32110<br>PRJEB9911	 [1]
Manech Tête Rousse	20	ERR3712282; ERR3712283; ERR3712284; ERR3712285; ERR3712286; ERR3712287; ERR3712288; ERR3712289; ERR3712290; ERR3712291; ERR3712292; ERR3712293; ERR3712294; ERR3712295; ERR3712296; ERR3712297; ERR3712298; ERR3712299; ERR3712300; ERR3712301	PRJEB35682	
Romane x Martinik Blackbelly	13	ERR3255915; ERR3255916; ERR3255917; ERR3255918; ERR3255919; ERR3988554; ERR3988555; ERR3988556; ERR3988557; ERR3988558; ERR3988559; ERR3988560; ERR3988561	PRJEB31930	
Cambridge	7	ERR1419201; ERR1419202; ERR1419203; ERR1419204; ERR1419205; ERR1419206; ERR1419207	PRJEB14098	
Vendéen	5	ERR4236129; ERR4236130; ERR4236131<br>SRR14934353; SRR14934354	PRJEB37460<br>PRJNA698548	[2]
Romane	4	ERS1205902; ERS1205903; ERR2818429; ERR2818430	PRJEB14418	[3]
Berrichon du Cher	3	ERS1205899; ERS1205900; ERS1205901	PRJEB14418	[3]
Noire du velay	2	ERR3828659; ERR3828660	PRJEB35553	[4]
Belclare	2	SRR14934360; SRR14935129	PRJNA698548	
Romanov	2	ERS1205904; ERS1205905	PRJEB14418	[3]
Suffolk	2	SRR14934357; SRR14934358	PRJNA698548	
Texel	2	SRR14934355; SRR14934356	PRJNA698548	
Martinik Blackbelly	1	ERR3255914	PRJEB31930	
Charollais	1	SRR14934359	PRJNA698548	
**Total**	**88**			

* LDHH6 heterozygous carrier

1. Rupp, R.; Senin, P.; Sarry, J.; Allain, C.; Tasca, C.; Ligat, L.; Portes, D.; Woloszyn, F.; Bouchez, O.; Tabouret, G.; et al. A Point Mutation in Suppressor of Cytokine Signalling 2 (Socs2) Increases the Susceptibility to Inflammation of the Mammary Gland While Associated with Higher Body Weight and Size and Higher Milk Production in a Sheep Model. *PLoS Genet* **2015**, *11*, e1005629, doi:10.1371/journal.pgen.1005629.
2. Fabre, S.; Chantepie, L.; Plisson-Petit, F.; Sarry, J.; Woloszyn, F.; Genet, C.; Drouilhet, L.; Tosser-Klopp, G. A Novel Homozygous Nonsense Mutation in ITGB4 Gene Causes Epidermolysis Bullosa in Mouton Vendéen Sheep. *Animal Genetics* **2020**, doi:https://doi.org/10.1111/age.13026.
3. Demars, J.; Cano, M.; Drouilhet, L.; Plisson-Petit, F.; Bardou, P.; Fabre, S.; Servin, B.; Sarry, J.; Woloszyn, F.; Mulsant, P.; et al. Genome-Wide Identification of the Mutation Underlying Fleece Variation and Discriminating Ancestral Hairy Species from Modern Woolly Sheep. *Molecular Biology and Evolution* **2017**, *34*, 1722–1729, doi:10.1093/molbev/msx114.
4. Chantepie, L.; Bodin, L.; Sarry, J.; Woloszyn, F.; Plisson-Petit, F.; Ruesche, J.; Drouilhet, L.; Fabre, S. Genome-Wide Identification of a Regulatory Mutation in BMP15 Controlling Prolificacy in Sheep. *Front. Genet.* **2020**, *11*, doi:10.3389/fgene.2020.00585.

Table S2. Semen parameters of dairy Lacaune rams from artificial insemination center.

Semen parameters*	LDHH6 non-carriers (+/+)	LDHH6 heterozygous carriers (m/+)
Volume (lsmean, mL)	1.77	1.67
Concentration (lsmean, x10^6spz/mL)	4.23	4.25
Motility (lsmean, score 0-5)	4.09	4.03
Proportion of collected semen used	0.80	0.76

*Data comprised 148,799 records during the 2010-2018 period. No significant genotype effect on semen parameters evaluated by linear model using the GLM procedure in the SAS software (version 9.4; SAS Institute Inc., Cary, NC) considering age at collection, season*year, moment of day collection (AM, PM), collection interval and LDHH6 status as fixed effects.

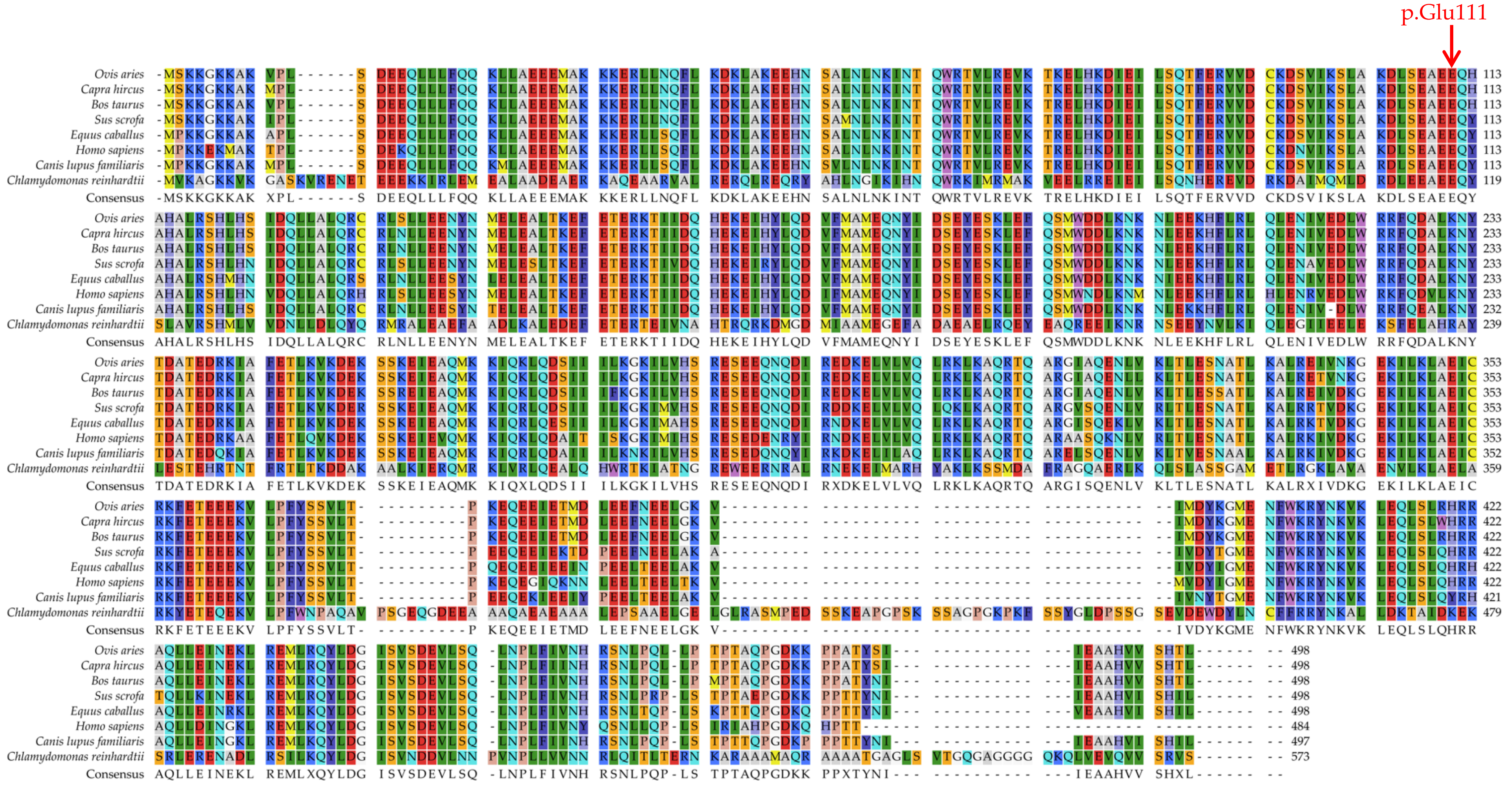

Figure S1. CCDC65 protein multiple sequence alignment.

Alignement of mammals (*Ovis aries* XP_004006438, *Capra hircus* XP_005680069, *Bos taurus* NP_001033255, *Sus scrofa* XP_020947250, *Equus caballus* XP_001504180, *Homo sapiens* NP_149115, *Canis lupus familiaris* XP_038294234) and green alga (*Chlamydomonas reinhardtii* DRC2_CHLRE) sequences was performed using MUSCLE algorythm in CLC Main Workbench 7.9.1 (Qiagen). All CCDC65 protein sequences are available at NCBI (https://www.ncbi.nlm.nih.gov/).

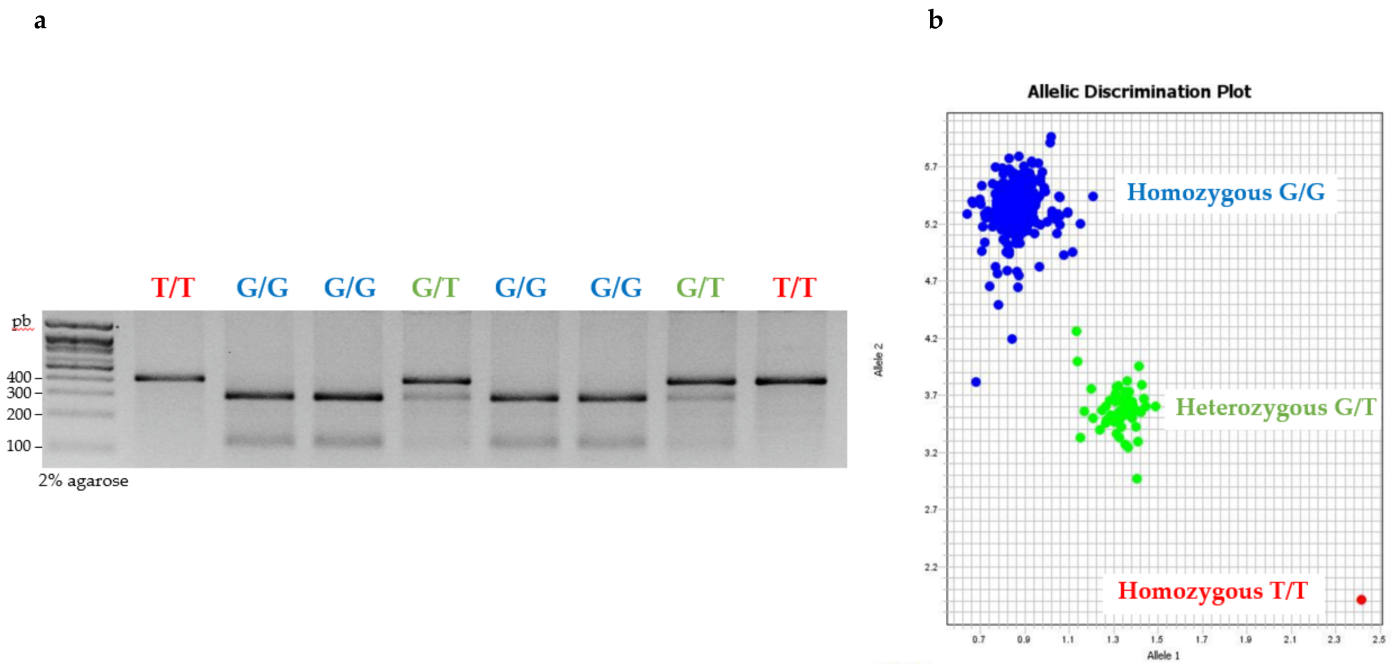

Figure S2. Representative genotyping results for c.521G>T variant in *CCDC65.*

(**a**) RFLP profiles after BsaJI digestion; and (**b**) Allelic discrimination plot from PACE analysis for homozygous carrier (T/T), heterozygous carrier (G/T) and non-carrier lambs (G/G) of the T variant allele.

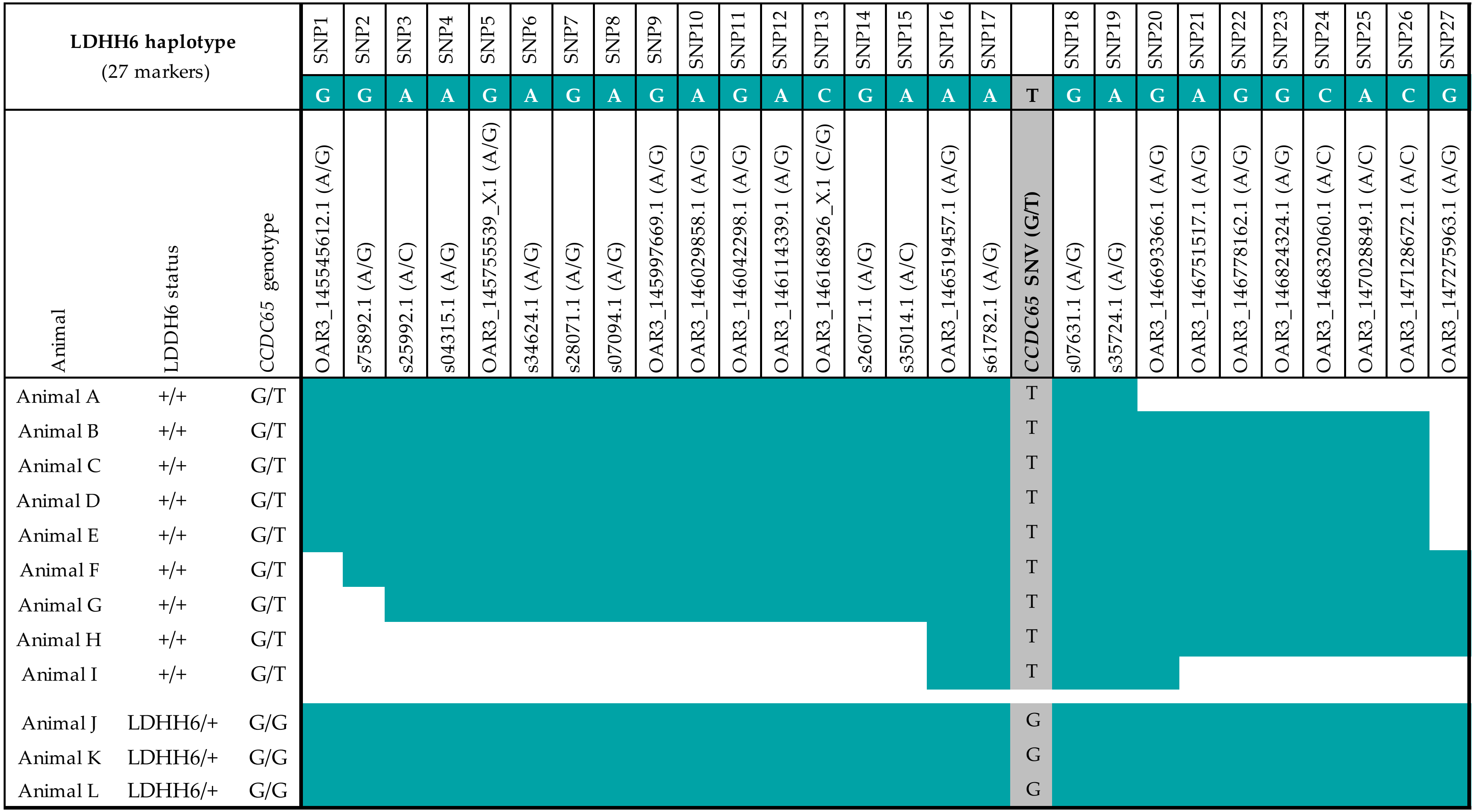

Figure S3. Haplotypes of the LDHH6 region for 12 animals showing mismatch with the *CCDC65* SNV.

LDHH6/+ and +/+ refer to heterozygous and non-carriers of LDHH6, respectively. The grey column represents the localization of the *CCDC65* variation (G>T) within the LDHH6 haplotype. For each animal, only the phase supposed to host the *CCDC65* T variant allele is represented. The blue color indicates the portion of local haplotype matching with the LDHH6 haplotype.
